# From SPINE to SPINE-2 complexes and beyond

**DOI:** 10.1016/j.jsb.2011.05.013

**Published:** 2011-08

**Authors:** Anastassis Perrakis, Susan Daenke, David I. Stuart, Joel L. Sussman

**Affiliations:** Department of Biochemistry, NKI, Plesmanlaan 121, 1066 CX Amsterdam, The Netherlands; Division of Structural Biology and Oxford Protein Production Facility, Wellcome Trust Centre for Human Genetics, University of Oxford, Roosevelt Drive, Oxford OX3 7BN, United Kingdom; Division of Structural Biology and Oxford Protein Production Facility, Wellcome Trust Centre for Human Genetics, University of Oxford, Roosevelt Drive, Oxford OX3 7BN, United Kingdom; Science Division, Diamond Light Source Ltd., Diamond House, Harwell Science and Innovation Campus, Didcot, Oxfordshire OX11 0DE, United Kingdom; Department of Structural Biology, Weizmann Institute of Science (ISPC), Rehovot 76100, Israel; The Israel Structural Proteomics Center, Faculty of Biochemistry, Weizmann Institute of Science, Rehovot 76100, Israel

It is about a decade since SPINE – Structural Proteomics IN Europe – was envisaged, set-up and submitted as an idea to the European Commission fifth framework programme. Our concepts were built on the paradigm of the USA-based structural genomics projects (http://www.nigms.nih.gov/psi), the Japanese initiatives (http://www.rsgi.riken.go.jp/rsgi_e), and some national efforts within Europe. At the same time, benefiting from the experience and technology development of the preceding projects, SPINE took an approach different from that of the mainstream of structural genomics: a major success of SPINE was to bring the cutting-edge high-throughput (HTP) technologies to biomedically relevant targets, not only facilitating the determination of no less than 308 novel structures, but also achieving a highly collaborative spirit and a culture of exchange of knowledge in technology.

SPINE-2 Complexes (S2C) was both a continuation of SPINE and a whole new venture in its own right. Having largely achieved the integration of HTP technologies at partner sites, S2C aimed to facilitate the study of macromolecular complexes in key areas of biological research (ubiquitin (de-)conjugation, cell cycle and apoptosis, synaptogenesis and neuronal signaling, kinases and phosphatases, transcription receptors and regulation, innate and acquired immunity, and mechanisms in viral infection). S2C also worked to advance ‘wet-lab’ technologies relevant to the study of challenging targets (e.g., production of multi-subunit complexes with an emphasis on eukaryotic systems, biophysical methods to characterize complexes, and library tools for HTP screening), and ‘dry-lab’ technologies (e.g., microcrystal handling, docking software, as well as data management, analysis and dissemination tools). This ambitious agenda was backed-up by the long standing interests of the participant labs in the corresponding research themes, and was supported by a shared enthusiasm for consolidation of technologies and conceptual approaches, which together allowed us to showcase the structural study of complex systems.

This special issue appears at a time at which Integrative Structural Biology is beginning to blossom. It seems, therefore, to be appropriate to take the opportunity to look back at what was achieved in S2C, and to present papers summarizing progress in key areas of biological interest, as well as on describing new technological achievements and benchmarking and extending existing methodologies.

In the Foreword of a special issue of Acta Crystallographica devoted to SPINE (vol. D62, part 10), Ray Stevens forecast beyond SPINE-2 Complexes (which at the time had just been funded) to SPINE-3 and beyond, a future that was never formally realized. It is likely, however, to be superseded by an even brighter future, largely as an outcome of SPINE and S2C: *Instruct* (http://www.structuralbiology.eu), via a coalition of biotechnology core centers in Europe, will provide access for European structural cell biologists to advanced instrumentation and to valuable expertise, thereby democratizing the ability to undertake technologically challenging research and increasing the competitiveness of European science. We hope that the research presented in this special issue, focusing on the work done within the SPINE networks, has helped to pave a bright European future for Structural Biology.

